# Hepatitis B virus genotypes prevalence in patients from hepatology services in Ceará, Brazil

**DOI:** 10.1590/0037-8682-0807-2020

**Published:** 2021-09-06

**Authors:** José Napoleão Monte Da Cruz, Lívia Melo Villar, Francisco Campello Do Amaral Mello, Elisabeth Lampe, Elodie Bomfim Hyppolito, José Milton De Castro Lima, Silvia Bomfim Hyppolito, Roberto Da Justa Pires, Larissa Deadame De Figueiredo Nicolete, Ivo Castelo Branco Coelho

**Affiliations:** 1 Universidade Federal do Ceará, Departamento de Patologia, Programa de Pós-Graduação em Patologia, Fortaleza, CE, Brasil.; 2 Fundação Oswaldo Cruz, Laboratório de Hepatites Virais, Rio de Janeiro, RJ, Brasil.; 3 Hospital São José, Fortaleza, CE, Brasil.; 4 Universidade Federal do Ceará, Hospital Universitário Walter Cantidio, Fortaleza, CE, Brasil.; 5 Universidade Federal do Ceará, Maternidade Escola Assis Chateaubriant, Fortaleza, CE, Brasil.; 6 Universidade Federal do Ceará, Departamento de Saúde Comunitária, Fortaleza, CE, Brasil.; 7 Universidade Internacional da Integração da Lusofonia Afro-Brasileira, Instituto de Ciências da Saúde, Redenção, CE, Brasil.

**Keywords:** Hepatitis B vírus, Genotype, Prevalence

## Abstract

**INTRODUCTION:**

Hepatitis B virus (HBV) infection is a public health problem; therefore, we aimed to report HBV genotypes in Ceará, Brazil.

**METHODS:**

A total of 103 HBsAg-positive samples were subjected to HBV genotyping and subgenotyping.

**RESULTS:**

The following genetic compositions of samples were found: F-54% (F2-83.33%), A-40% (A1-65%), D-6%, C2-1%, E-1%, and G-1%.

**CONCLUSIONS:**

Some genotypes are only prevalent in certain parts of the world; however, the State of Ceará is a hub for migration and has one of the most important liver transplantation centers in Brazil, which can explain the prevalence of the F genotype.

It is estimated that more than 257 million individuals are chronically infected with hepatitis B virus (HBV), and many factors, such as HBV genotype, are known to influence disease progression^1^. To date, 10 HBV genotypes have been identified across different geographic regions. A greater understanding of the relationship between HBV genotypes, progression of hepatitis B, and clinical outcomes have been developed over time^2^. This study aimed to describe the prevalence of HBV genotypes and subgenotypes in the state of Ceará, Brazil. 

Patients with chronic hepatitis B were invited to participate in the study with approval from the Ethics Committee of the Assis Chateaubriant University Maternity (opinion no. 084/09). A total of 103 patients were recruited from the Public Health Central Laboratory of Ceará (LACEN-CE). Serum samples were subjected to HBV DNA detection by polymerase chain reaction using primers and thermal cycling conditions as described by Mallory et al. (2011), without modifications^3^. The fragment used in our analysis had 869 nucleotides, representing 27% of the HBV genome, where it was possible to obtain enough genetic information to distinguish and classify the viral strains found in subgenotypes, as represented in Figure 1. This method also generates electropherograms that can be evaluated for the main drug resistance-associated mutations, including rtL180M and rtM204I/V, used for genotype G. Positive samples were sequenced using the Sanger method^4^. Sequences were edited and aligned using MEGA X^5^. To align these sequences, 66 standard reference sequences of different HBV genotypes (A-J) were obtained from Genbank^6^. The phylogenetic tree was constructed using the maximum-likelihood method (1000 bootstrap replicates) under the model of nucleotide substitution GTR+G+I, which was selected as the best-fit model by the j-Model test^7^. Among the 103 individuals tested, most belonged to genotype F (n=54; 52.4%) (F1 n=1; F2 n=45; F4 n=8), followed by genotypes A (n=40; 38.8%) (A1, n=26; A2, n=14), G (n=1; 1.0%), D (n=6; 5.8%) (D2 n=4; D3 n= 2), E (n=1; 1.0%), and C2 (n=1; 1.0%) (Figure 1). 

**FIGURE 1:**
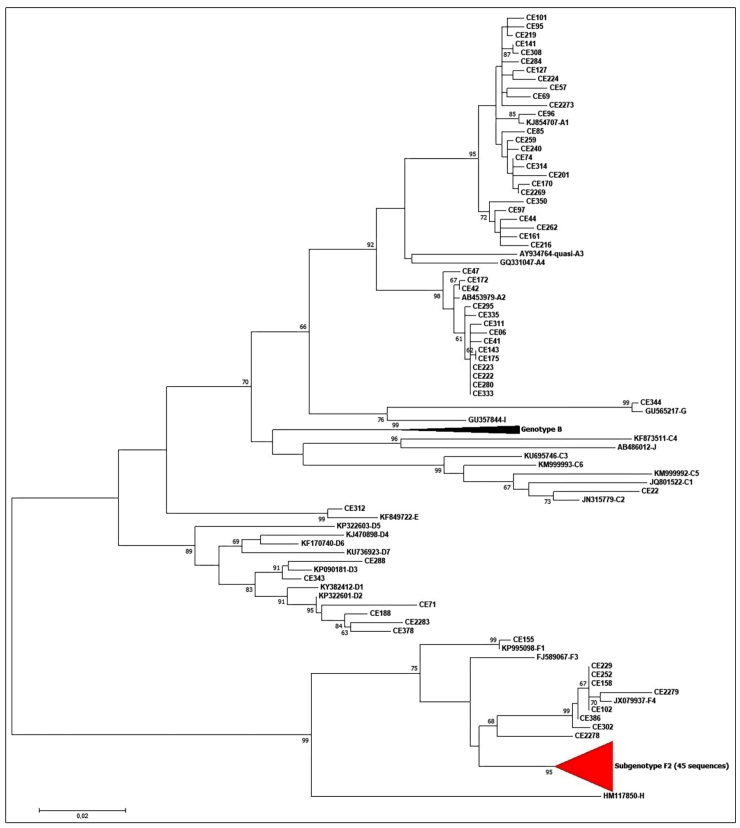
The Genetic grouping of 103 samples from patients with chronic HBV infection was amplified and the generated fragment of 869 nucleotides was used in our phylogenetic analisys. The reference subgenotype strains are represented by the Genbank numbers as follows: A1/Brazil (KJ854707); quasi-A3/Gambia (AY934764); A2/Japan (AB453979); G/Neitherlands (GU565217); C4/Australia (KF873511); C1/Thailand (JQ801222); E/Angola (KF84972); D3/Brazil (KP090181); D2/India (KP322601); F4/Argentina (JX079937); F2/Brazil (KT896494) and Venezuela (KP 995115); F1/Venezuela (KP995098). All bootstrap values were represented.

This study found a high prevalence of genotype F2 in Ceará, followed by the A1 genotype, which was previously reported by Lampe et al. (2017)^8^. Based on the most prevalent results, a recent study suggests that HBV/F2 in the Brazilian HBV chronic population is related to clades from Venezuela^9^. Despite the origin of circulating genotypes, the HBV/F2 prevalence should be examined more carefully and better investigated in future studies. Pujol (2020)^10^ mentions that mutations in the basal core promoter may be more frequent in some subgenotypes, such as F1b and F2, than in other American subgenotypes or the H genotype. The F1b genotype, and most likely the F2 genotype, may be associated with a severe presentation of liver disease as opposed to a more benign course for subgenotype F4 and genotype H^10^. 

Limeres et al. (2019) demonstrated that HBsAg, produced from strains of wild-type F1b and F4 subgenotypes, and the G145R mutant may have detection rates significantly different from those obtained for genotype A. The influence of the high genetic variability of HBV on the sensitivity of serological and molecular tests has received little attention so far. As mentioned above, one of the main sources of variability is related to viral genotypes and subgenotypes. In this article, the authors noted that the F1b subgenotype had a major impact on the sensitivity of the immunoassays tested. The same authors provided disturbing data for the positive detection of HBsAg with F genotype samples and suggested that the incorporation of an F-derived genotype should be evaluated in areas where it is endemic. Finally, the authors suggest that these changes in genotypes and subgenotypes found in HBsAg may affect the effectiveness of vaccination, which is performed with antigens on the surface of the HBV virus^11^. 

Genotype A is the most prevalent in Brazil, however there are differences in its introduction throughout the country. While subgenotype A2 is linked to European colonization, HBV/A1 could have been introduced in Brazil by both African slaves and Asian merchants^9^. A comparative phylogeographic analysis was not carried out with other A1 subgenotypes, however, the State of Ceará has a close relationship with the abolitionist history of Brazil, as the city of Redenção, located just 55 km from Fortaleza, was the first in Brazil to free the slaves. This historic event took place on March 25, 1884. In addition, the country has an intimate relationship with the African continent, due to the presence of an international university located in this city^12^ ^,^ ^13^. 

The association between Ceará´s population and Africa can also explain the presence of genotype E found in our study. The most recent work on this genotype reaffirms that its circulation is concentrated in West Africa and, when outside that region, belongs to people of African descent^14^. Our data suggest that this genotype may be circulating in the Brazilian population, and its prevalence should be further investigated in other studies, as HBV/E has been clinically characterized with significantly higher viral loads and chronicity rates and a milder clinical manifestation^14^ ^,^ ^15^. 

The third most common genotype in the present study was D. This is usually more prevalent than genotype F in several regions of the country, and in the South, it is more prevalent than genotype A^9^ ^,^ ^16^, demonstrating that the European influence, especially the Italian one, may be less influential in the transmission of HBV within the state of Ceará. Examining the migratory flow proposed by Bomtempo (2019)^17^, it is noted that Europeans continue to prefer to immigrate to the states in the South and Southeast regions of Brazil, with Ceará and Bahia receiving a smaller flow of these immigrants^17^. 

The same author, however, highlights the increased migratory flow, with the arrival of Chinese and Koreans to the state of Ceará, which may explain the presence of the C2 genotype identified in our study. It is worth mentioning that an interesting and relevant study was recently published that evaluated the worldwide distribution of HBV genotypes. The study demonstrates that China, Southeast Asia, and Australia have a very high prevalence of genotype C and that it is responsible for the majority of chronic infections around the world^18^. In Brazil, HBV/C has already been reported to have a 13.1% prevalence in São Paulo and to be of Asian origin, which suggests that this genotype, when found in Brazil, is also part of a migratory flow^19^. 

Finally, this study also detected the presence of a sample with genotype G, as previously described by Lampe et al. (2017). Monoinfection by HBV genotype G was confirmed by submitting the extracted viral DNA to commercial assay "INNO-LiPA® HBV Genotyping" (Fujirebio Europe N.V., Zwijnaarde, Belgium), a highly sensitive line probe assay capable of identifying minor viral subpopulations, that is, the occurrence of co-infection with different HBV genotypes^8^. In the same sample, the occurrence of an L180M + rtM204V mutation was also observed, which is responsible for the resistance to lamivudine and telbivudine (LdT). Although it is not used in the treatment of HBV patients, the discovery of this mutation corroborates the findings previously described by Araújo et al. (2020), who demonstrated that this finding is strongly associated with the G genotype^20^. 

As HBV/G is rarely found without association with genotype A in our territory, we decided to observe whether this mutation would be present in our sample. Interestingly, genotype G is associated with men who have sex with men co-infected with HIV and may suggest an association of this sample with this risk group^20^. Araújo et al. (2020) suggested that Lamivudine (LAM) is widely used in the treatment of HBV and HIV viruses. This can lead to stronger selective pressure due to the more frequent use of LAM in isolates of the G genotype than in other genotypes, resulting in higher frequencies of LAM-resistant mutants and LdT due to cross-resistance^20^. However, the same authors observed these findings in other genotypes and demonstrated that antiviral resistance mutations were not relevant in the F genotype, which is the predominant HBV genotype in our study.

These authors suggest that the mobility of populations contributed to the introduction of genotypes in a region. Historically, a great migratory flow from Ceará to the North region of the country due to labor opportunities. The occurrence of this migratory flow and the return to Ceará cities may be responsible in terms of the occurrence of this pathogenicity, different from the original population. Another explanation could be that the samples were obtained were from patients coming from healthcare assistance in Ceará and those that were on the waitlist for liver transplantation, since this state is one of the most important liver transplantation centers in Brazil^21^. Although this study was conducted to confirm the circulation of HBV genotypes and sub-genotypes, it is important to reinforce that epidemiologic studies are needed to elucidate the reasons and importance of the genotypic pattern found. The present study’s findings regarding the prevalence of genotypes contributes to defining the profile of circulating viruses in the State of Ceará, assisting in the strategic actions of the Ministry of Health. 
